# A Database of Wing Diversity in the Hawaiian *Drosophila*

**DOI:** 10.1371/journal.pone.0000487

**Published:** 2007-05-30

**Authors:** Kevin A. Edwards, Linden T. Doescher, Kenneth Y. Kaneshiro, Daisuke Yamamoto

**Affiliations:** 1 Department of Biological Sciences, Illinois State University, Normal, Illinois, United States of America; 2 Center for Conservation Research and Training, University of Hawaii at Manoa, Honolulu, Hawaii, United States of America; 3 Graduate School of Life Sciences, Tohoku University, Sendai, Japan; Ecole Normale Supérieure de Lyon, France

## Abstract

**Background:**

Within genus *Drosophila*, the endemic Hawaiian species offer some of the most dramatic examples of morphological and behavioral evolution. The advent of the *Drosophila grimshawi* genome sequence permits genes of interest to be readily cloned from any of the hundreds of species of Hawaiian *Drosophila*, offering a powerful comparative approach to defining molecular mechanisms of species evolution. A key step in this process is to survey the Hawaiian flies for characters whose variation can be associated with specific candidate genes. The wings provide an attractive target for such studies: Wings are essentially two dimensional, and genes controlling wing shape, vein specification, pigment production, and pigment pattern evolution have all been identified in *Drosophila*.

**Methodology/Principal Findings:**

We present a photographic database of over 180 mounted, adult wings from 73 species of Hawaiian *Drosophila*. The image collection, available at FlyBase.org, includes 53 of the 112 known species of “picture wing” *Drosophila*, and several species from each of the other major Hawaiian groups, including the modified mouthparts, modified tarsus, *antopocerus*, and *haleakalae* (fungus feeder) groups. Direct image comparisons show that major wing shape changes can occur even between closely related species, and that pigment pattern elements can vary independently of each other. Among the 30 species closest to *grimshawi*, diverse visual effects are achieved by altering a basic pattern of seven wing spots. Finally, we document major pattern variations within species, which appear to result from reduced diffusion of pigment precursors through the wing blade.

**Conclusions/Significance:**

The database highlights the striking variation in size, shape, venation, and pigmentation in Hawaiian *Drosophila*, despite their generally low levels of DNA sequence divergence. In several independent lineages, highly complex patterns are derived from simple ones. These lineages offer a promising model system to study the evolution of complexity.

## Introduction

Nearly 1000 species of *Drosophila* are endemic to Hawaii, yet current evidence suggests they arose from a single introduction to the Hawaiian Island chain roughly 26 million years ago [1]–[6]. The “picture wing” group consists of 112 known species, most of which are quite distinct from each other in morphology, pigmentation, and behavior, even when they are separated by ∼0.5 million years of divergence (the age of the island of Hawaii [3], [7]). This explosive adaptive radiation is now known to have occurred with relatively little change in DNA sequence [8]–[10]. These factors make the Hawaiian *Drosophila* an important model system for analysis of evolutionary processes at the species level.

The *Drosophila grimshawi* genome has been sequenced [11], [12], providing a major new entry point into genomic and molecular genetic analyses of the Hawaiian flies. High levels of similarity to the *grimshawi* sequence should permit the amplification of nearly any gene of interest from a range Hawaiian species. Identified sequence differences can then be correlated with phenotypic variations among the species, providing insights into molecular mechanisms of evolution. To make the most of this opportunity, it is important for researchers to have access to uniformly collected phenotypic data from numerous species. The data can be used to identify characters that show interesting patterns of variation, and for which candidate genes can be hypothesized. The *Drosophila* wing is an attractive target for such candidate-based studies, since wing development has been analyzed in great detail in *D. melanogaster* [13]–[16], and genes controlling wing shape [17]–[19] and pigmentation [20]–[23] have been identified.

Wing pigment spots occur in highly reproducible, species-specific, two-dimensional patterns, and their genetics and development are beginning to be understood. True et al. [21] found that wing spot patterns have two main components: a vein-independent “prepattern” formed during wing development prior to eclosion, and vein-dependent melanization that forms after eclosion. In species such as *grimshawi*, the prepattern is faintly visible upon eclosion, marked by an arrangement of dark versus light wing hairs. In the first day or two after eclosion, pigment precursors travel through the wing veins and diffuse into the intervein regions, allowing further darkening of the cuticle into clearly visible spots. In this model, the spots must contain localized enzymes that are waiting to convert the precursors to melanins. This enzyme prepattern is most likely formed by localized expression of pigmentation genes in response to the wing's basic patterning machinery. Wing spot evolution would then involve changes in either the upstream patterning genes, or the downstream pigmentation genes. Changes in patterning genes would tend to be pleiotropic, altering other features of the wing, so this explanation is unlikely when only pigment changes are observed. Thus, the favored explanation is that mutations occur in the *cis*-regulatory regions of the pigmentation genes, bringing them under control of existing, region-specific activators or repressors [23]. Such mutations could be very selective, altering only portions of the original expression pattern. A related possibility is that a “dedicated” transcription factor controls one or more pigmentation genes, and this transcription factor is the target of regulatory mutations [24].

Studies of the *yellow* locus have provided multiple examples of regulatory mutations controlling the evolution of wing spots. The Yellow protein is required to pigment the cuticle, and ectopic Yellow causes dark pigmentation in a wild type background. This Yellow-dependent pigmentation is strongly enhanced by removal of Ebony protein (beta-alanyl-dopamine synthase) [22]. Yellow and Ebony promote separate branches of the pathway from dopa to variously colored pigments [23]. The *yellow* and *ebony* genes have been co-opted during evolution to produce wing spots: a male-specific wing spot in *D. biarmipes* is presaged by increased Yellow and decreased Ebony protein levels, and the extent of the spot is controlled in part by *engrailed* regulation of *yellow* via a novel *cis*-regulatory element [25]. The expression of Yellow protein in presumptive wing spots has been gained and lost multiple times in the evolution of genus *Drosophila*, and *yellow* has at least two distinct regulatory elements that can be co-opted to produce spots [26].

These studies provide the framework required to understand the evolution of complex pigment patterns in the Hawaiian *Drosophila*. Unfortunately, these pigment patterns have not been photographically documented in the literature, apart from a few sporadic examples (e.g., True et al. [21]). Intact flies have been photographed [27], but those pictures cannot be used for quantitative analysis or direct comparisons of wings between species. The original species descriptions (e.g. [28]–[32]) employ hand drawings, which are inherently limited in their ability to capture subtle variations in pigment color and density. These publications can also be difficult to obtain (though scanned versions can be found at the Japan Drosophila Database on Taxonomy, www.dgrc.kit.jp/∼jdd).

Here we present a photo database documenting the wings of 73 Hawaiian *Drosophila* species. Mounted wings were digitally photographed under uniform conditions to allow for comparisons between specimens, and the photos have been made available for download at FlyBase [33]. This collection highlights the astonishing diversity of the Hawaiian flies, first noted by Grimshaw in 1901 [34], and we hope it will inspire the fly community to leverage the *grimshawi* genome to gain further molecular insights into morphological evolution.

## Results and Discussion

**Figure 1:**
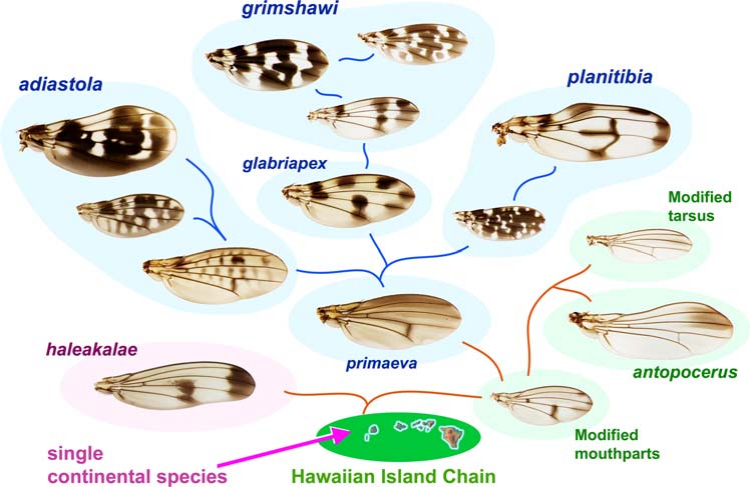
Overview of relationships among major species groups and picture wing subgroups. Schematic based on chromosomal inversions, DNA sequence data, and morphology [1], [4], [40], [41]. Arrow indicates the proposed single introduction of *Drosophila* to an island west of Kauai. Blue backgrounds, picture wing subgroups; green backgrounds, modified mouthparts, modified tarsus, and *antopocerus* groups; pink background, *haleakalae*/fungus feeder group. Lines schematically indicate consensus phylogenetic relationships. Examples of increasing pattern complexity in the *adiastola* and *grimshawi* subgroups are shown. See Figs. 2–7 for species names. Hawaii map courtesy of geology.com and mapresources.com.

The endemic Hawaiian *Drosophila* arose from an introduction of a continental species to an island (now subsided) that predates Kauai, the oldest of the current high islands [2]. These flies diversified into several major species groups; Fig. 1 provides an overview of the relationships among the groups discussed here.

**Figure 2:**
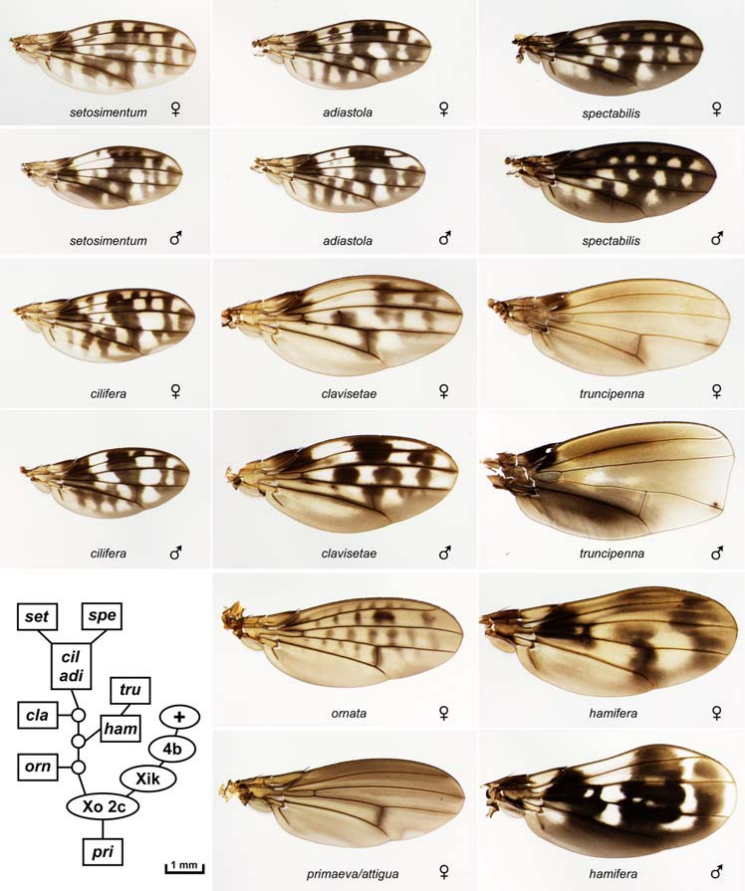
The *adiastola* and *primaeva*/*attigua* subgroups of picture wing species. *D. adiastola, cilifera, clavisetae, hamifera, setosimentum, spectabilis*, and *truncipenna* are shown as sexually dimorphic pairs, *ornata* and *primaeva*/*attigua* as single examples. In all figures, anterior is up and proximal is to the left. Inset, chromosome inversion-based lineage for the species shown (see text).

Over 180 different wings were photographed from 73 species of field-caught or lab-reared Hawaiian *Drosophila*. The original photographs and the montages are available in the Hawaiian Drosophila Wing Database at FlyBase [33]. Table 1 lists all the species available in the image database. In many cases, the database includes multiple wings per species; in this paper, the single most intact wing from each species is shown (Figs. 2–7). When both male and female wings are available, and sexual dimorphism is apparent, both sexes are shown; the most dramatic cases of dimorphism occur in the *adiastola* subgroup of picture wing species (Fig. 2). We have attempted to organize the figures based on established species groupings: the photos tend to be arranged phylogenetically and thus are not alphabetical. The full species descriptions, phylogenies, and behavioral and ecological data have been previously reported and are beyond the scope of this paper [1], [7], [8], [10], [28]–[32], [35]–[38].

**Figure 3 pone-0000487-g003:**
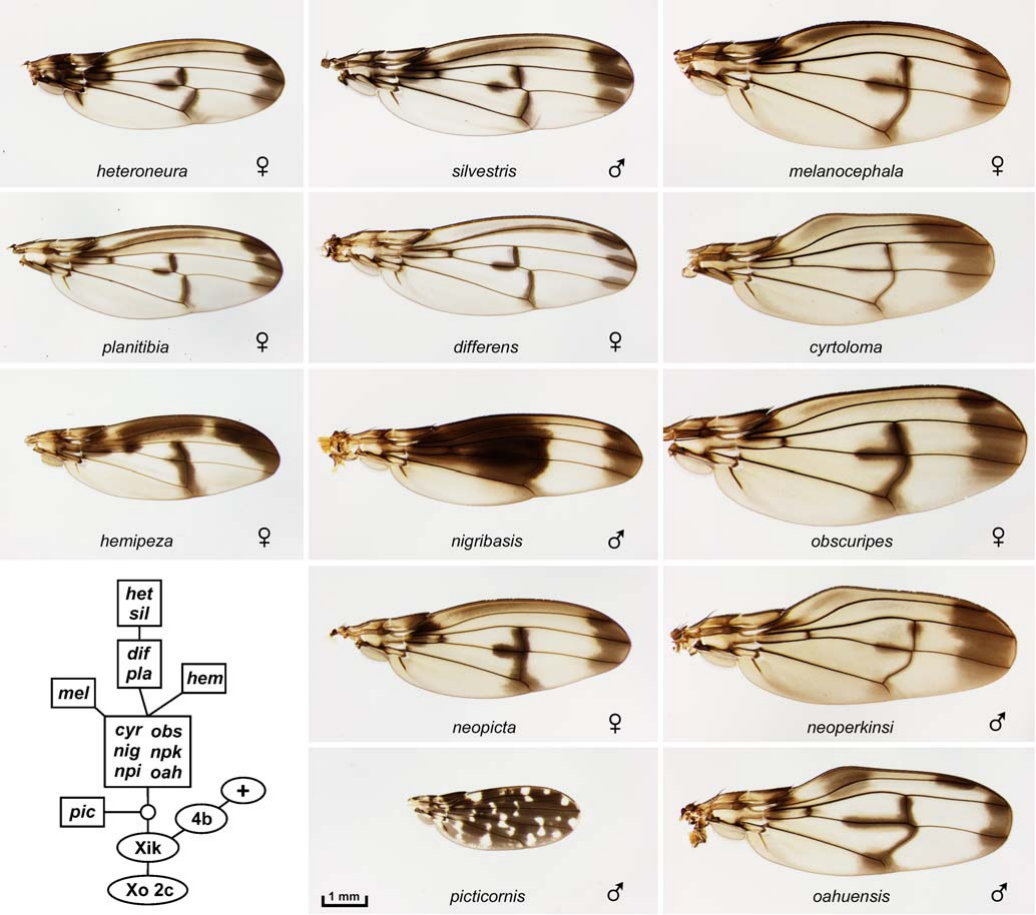
The *planitibia* subgroup of picture wing species[7]. The *planitibia* complex: *differens, hemipeza, heteroneura, planitibia*, and *silvestris*. The *neopicta* complex: *neopicta* (*npi*) and *nigribasis*. The *cyrtoloma* complex (right column): *cyrtoloma, melanocephala, neoperkinsi* (*npk*), *oahuensis*, and *obscuripes*. The *picticornis* complex: *picticornis*. Inset, chromosome inversion-based lineage for the species shown (see text). The six species in the large box arose from an ancestral population that was polymorphic for the inversions that now differ among these species; see Carson [41] for details.

**Figure 4 pone-0000487-g004:**
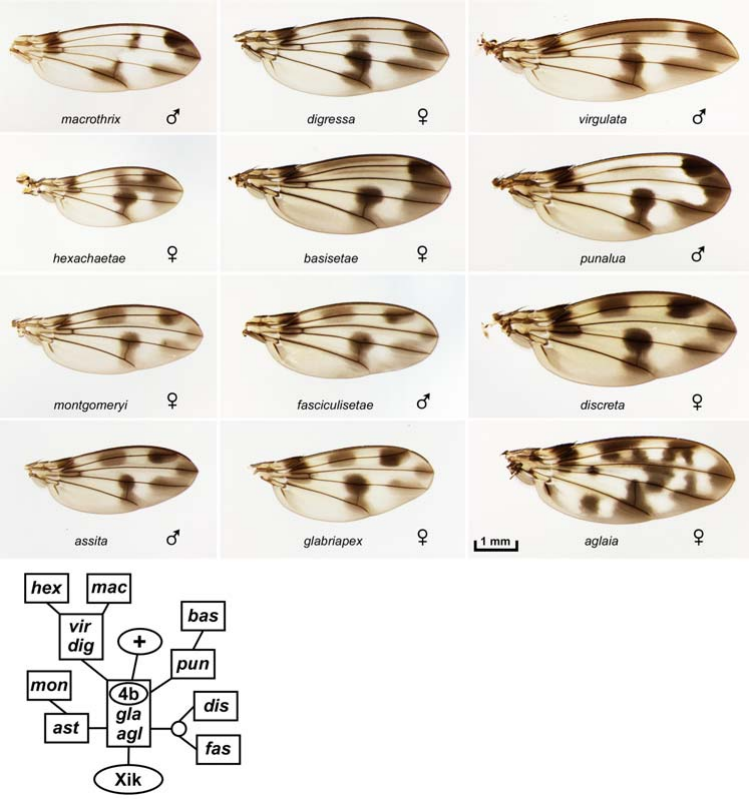
The *glabriapex*/4b subgroup of picture wing species: *D. aglaia, assita, basisetae, digressa, fasciculisetae, glabriapex, hexachaetae, macrothrix, montgomeryi, punalua*, and *virgulata*. Lower, chromosome inversion-based lineage for the species shown (see text).

**Figure 5 pone-0000487-g005:**
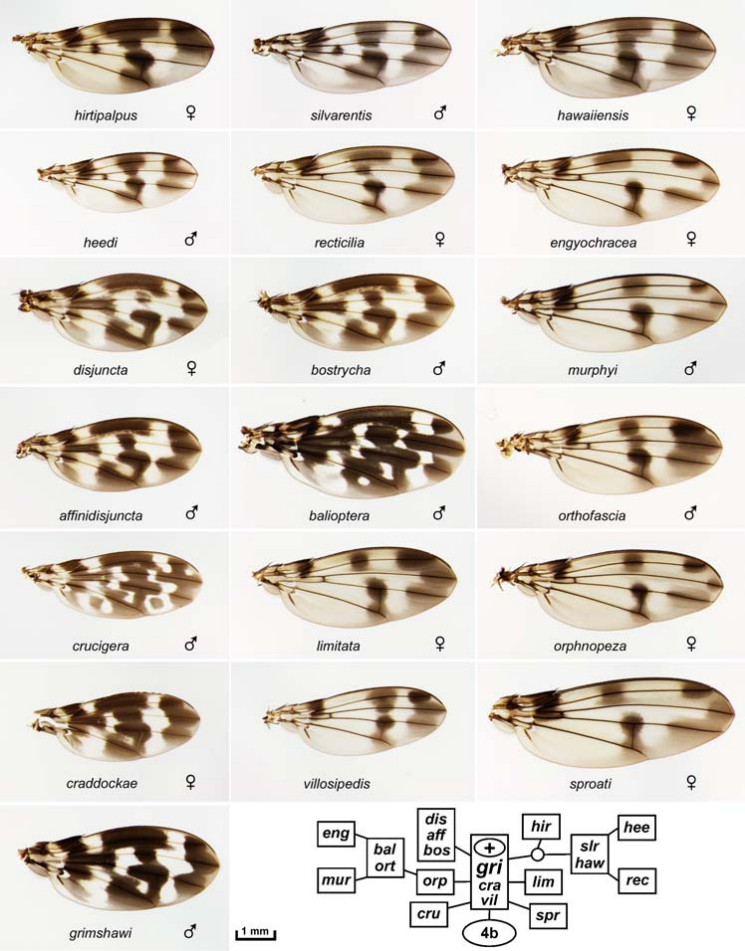
The *grimshawi*/4b+ subgroup of picture wing species: *D. affinidisjuncta, balioptera, bostrycha, craddockae, crucigera, disjuncta, engyochracea, grimshawi, hawaiiensis, heedi, hirtipalpus, limitata, murphyi, orphnopeza, orthofascia, recticilia, silvarentis, sproati*, and *villosipedis*. Inset, chromosome inversion-based lineage for the species shown (see text).

**Figure 6 pone-0000487-g006:**
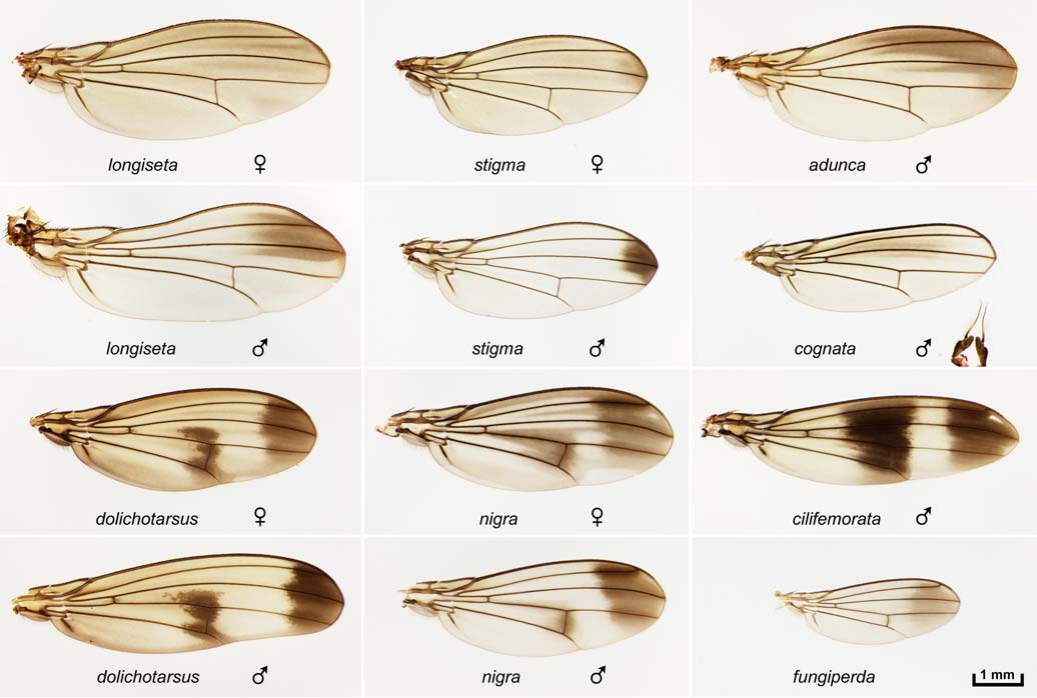
The *antopocerus* and *haleakalae*/fungus feeder groups. Upper six panels, *antopocerus* group species *D. longiseta, stigma, adunca*, and *cognata*. Sexual dimorphism is shown for *longiseta* and *stigma*. The extended male antennal structures, characteristic of the *antopocerus* group, can be seen co-mounted with the *cognata* wing. Lower six panels, *haleakalae*/fungus feeder group: *dolichotarsus, nigra, cilifemorata*, and *fungiperda*. Sexual dimorphism is shown for *dolichotarsus* and *nigra*.

**Figure 7:**
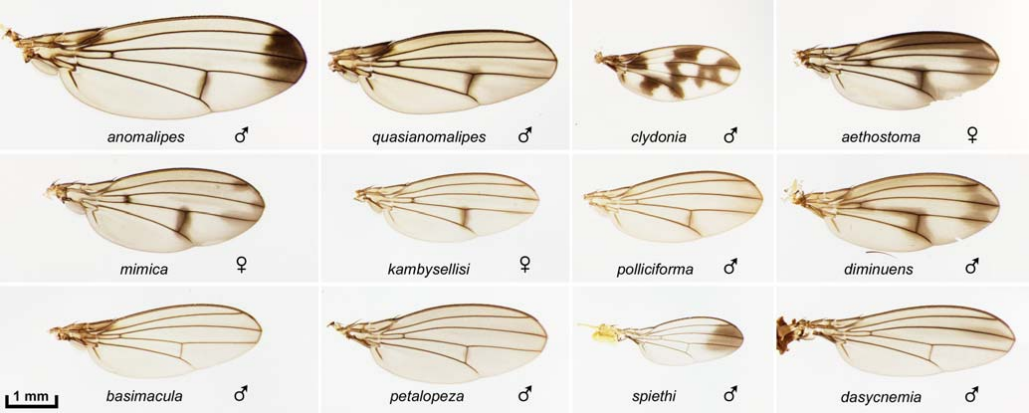
Other non-picture wing species. The *anomalipes* group: *anomalipes* and *quasianomalipes*. “Modified mouthparts group”: *clydonia, aethostoma, mimica, kambysellisi, polliciforma*, and *diminuens*. The ornate pattern of *clydonia* is rare among the small, non-picture wing species. The curved L3 vein in *clydonia* is a characteristic of the species [28]. “Modified tarsus group”: *basimacula, petalopeza, spiethi*, and *dasycnemia*.

**Table 1:** Species in the Hawaiian Drosophila Wing Database

Species	Island	group	Figure
*adiastola*	Maui	*adiastola*	2
*adunca*	Maui	*antopocerus*	6
*aethostoma*	Kauai	mod. mouthparts	7
*affinidisjuncta*	Maui	*grimshawi*	5
*aglaia*	Oahu	*glabriapex*	4
*anomalipes*	Kauai	*anomalipes*	7
*assita*	Hawaii	*glabriapex*	4
*balioptera*	Molokai	*grimshawi*	5
*basimacula*	Kauai	mod. tarsus	7
*basisetae*	Hawaii	*glabriapex*	4
*bostrycha*	Molokai	*grimshawi*	5
*cilifemorata*	Maui	fungus feeder	6
*cilifera*	Molokai	*adiastola*	2, 8
*clavisetae*	Maui	*adiastola*	2
*clydonia*	Maui	mod. mouthparts	7
*cognata*	Hawaii	*antopocerus*	6
*craddockae*	Kauai	*grimshawi*	5
*crucigera*	Oahu	*grimshawi*	5
*cyrtoloma*	Maui	*planitibia*	3
*dasycnemia*	Hawaii	mod. tarsus	7
*differens*	Molokai	*planitibia*	3
*digressa*	Hawaii	*glabriapex*	4
*diminuens*	Hawaii	mod. mouthparts	7
*discreta*	Maui	*glabriapex*	4
*disjuncta*	Maui	*grimshawi*	5
*dolichotarsus*	Maui	fungus feeder	6
*engyochracea*	Hawaii	*grimshawi*	5, 8
*fasciculisetae*	Maui	*glabriapex*	4
*fungiperda*	Hawaii	fungus feeder	6
*glabriapex*	Kauai	*glabriapex*	4
*grimshawi* [G1*]	Maui	*grimshawi*	5
*hamifera*	Maui	*adiastola*	2
*hawaiiensis*	Hawaii	*grimshawi*	5
*heedi*	Hawaii	*grimshawi*	5
*hemipeza*	Oahu	*planitibia*	3
*heteroneura*	Hawaii	*planitibia*	3
*hexachaetae*	Oahu	*glabriapex*	4
*hirtipalpus*	Maui	*grimshawi*	5
*kambysellisi*	Hawaii	mod. mouthparts	7
*limitata*	Maui	*grimshawi*	5
*longiseta*	Molokai	*antopocerus*	6, 8
*macrothrix*	Hawaii	*glabriapex*	4
*melanocephala*	Maui	*planitibia*	3
*mimica*	Hawaii	mod. mouthparts	7
*montgomeryi*	Oahu	*glabriapex*	4
*murphyi*	Hawaii	*grimshawi*	5
*neoperkinsi*	Molokai	*planitibia*	3
*neopicta*	Maui, Molokai	*planitibia*	3
*nigra*	Maui	fungus feeder	6
*nigribasis*	Oahu	*planitibia*	3
*oahuensis*	Oahu	*planitibia*	3
*obscuripes*	Maui	*planitibia*	3
*ornata*	Kauai	*adiastola*	2
*orphnopeza*	Maui	*grimshawi*	5, 8
*orthofascia*	Maui	*grimshawi*	5
*petalopeza*	Maui	mod. tarsus	7
*picticornis*	Kauai	*planitibia*	3
*planitibia*	Maui	*planitibia*	3
*polliciforma*	Hawaii	mod. mouthparts	7
*primeava* or *attigua*	Kauai	*primaeva*	2
*punalua*	Oahu	*glabriapex*	4
*quasianomalipes*	Kauai	*anomalipes*	7
*recticilia*	Maui	*grimshawi*	5
*setosimentum*	Hawaii	*adiastola*	2
*silvarentis*	Hawaii	*grimshawi*	5
*silvestris*	Hawaii	*planitibia*	3
*spectabilis*	Molokai	*adiastola*	2
*spiethi*	Kauai	mod. tarsus	7
*sproati*	Hawaii	*grimshawi*	5, 8
*stigma*	Molokai	*antopocerus*	6
*tanythrix*	Hawaii	*antopocerus*	8
*truncipenna*	Maui	*adiastola*	2
*villosipedis*	Kauai	*grimshawi*	5
*virgulata*	Maui	*glabriapex*	4

All species are genus *Drosophila* [57]. *G1 is the stock from which the genome sequence was derived.

### The picture wing flies

We obtained specimens of 53 of the 112 species in the picture wing group, including representatives of all major lineages. Notably, the US Federal endangered species list includes a total of just 51 insect species, and 11 of these are Hawaiian picture wing *Drosophila*. Five of these endangered species are included here: *heteroneura, differens*, and *hemipeza* (Fig. 3); *aglaia* and *montgomeryi* (Fig. 4). All were collected prior to the endangered species designation.

The picture wing group is divided into four major subgroups named for representative species: *adiastola* (Fig. 2), *planitibia* (Fig. 3), *glabriapex* (Fig. 4) and *grimshawi* (Fig. 5). A nearly complete lineage of the picture wing flies was determined by H.L. Carson, who used polytene chromosome banding patterns to map chromosomal inversions in each species [1], [39]–[41]. Carson's inversion tree is highly congruent with molecular phylogenies of the picture wings [42]. In order to provide some phylogenetic context for comparing the wings, we have reproduced the chromosomal lineages of the species that are shown in each of the picture wing figures (insets in Figs. 2–5). *D. grimshawi* is the arbitrarily chosen chromosomal standard (+). Each box represents a unique inversion genotype or karyotype present in the designated species (abbreviated to 3 letters). Circles represent inversion genotypes that do not match any species in the database; these are only included when they constitute nodes in the tree. The actual inversion names have been omitted for simplicity; see Carson [40] for complete genotypes. The chromosome map for a given species can be derived by adding all the inversions along the path to the standard, “+”. The ovals indicate three key sets of inversions, designated Xo 2c; Xik; and 4b; that uniquely define each picture wing subgroup. Specifically, the *grimshawi* subgroup lacks these inversions (since *D*. *grimshawi* is the standard); the *glabriapex* subgroup has 4b, the *planitibia* subgroup has Xik and 4b, and the *adiastola* and *primaeva* subgroups have Xo 2c, Xik, and 4b. Relationships among the four subgroups can be obtained by connecting the trees at these points, as summarized in Fig. 1. Note that branch lengths are arbitrary, since the number of inversions is not necessarily proportional to the time since divergence. Chromosomal trees are also inherently unrooted; this tree is rooted at *primaeva* based on DNA and biogeographic evidence [7], [8], [40].

Fig. 2 shows eight of the 16 members of the *adiastola* subgroup. These species are particularly notable for the intricate and subtly graded pigment patterns of the wings. In addition, much of this group shows pronounced sexual dimorphism, and so Fig. 2 includes male/female pairs for 7 species. The group's wing patterns are quite diverse. A third crossvein appears in *clavisetae* (and *neoclavisetae*, not shown), likely as an adaptation that provides mechanical support for larger wings. This adaptation arose independently in the *planitibia* subgroup (below)[43]. In *spectabilis*, the pigment spots are expanded and fused, giving the appearance of a black wing with light spots. The most extreme wing shape change in this collection (and perhaps in the genus) is seen in *truncipenna*, in which the male wings are blunted at the tips giving a nearly rectangular appearance. The female wing is slightly blunted as well, but the selection pressure on this phenotype appears to be focused on the males. The *hamifera* wing is perhaps the most divergent overall, with an exceptional combination of large size, distorted shape, and complex, dimorphic pigmentation. The males and females share a dark spot over the proximal part of longitudinal veins L2–4, but the rest of their patterns appear to be almost completely unrelated.

Fig. 2 includes a *primaeva*/*attigua* specimen; these two species are considered to form their own subgroup at the base of the picture wing clade (Fig. 1) [8], [40], [42]. The distinction between *primaeva* and *attigua* could not be made in this specimen since it was female.

Fig. 3 shows 13 of the 17 *planitibia* subgroup species (see recent phylogenetic analysis [7]). The group features the well-studied “hammerhead” species *heteroneura* and sister species *silvestris* (see Boake et al. [44] and refs therein). Flies of this group are exceptionally large, and this size increase is correlated with the appearance of a third crossvein in most species. The extra crossvein is usually aligned with the standard posterior crossvein, but it is shifted proximally in the closely related species complex *heteroneura, silvestris, planitibia* and *differens*. The subgroup is also known for using wing vibrations to produce complex courtship songs, and this behavior may be related to the unusual wing shapes of some species (e.g., *cyrtoloma* [45]). The *picticornis* wing is quite divergent, being mostly pigmented with numerous light spots; this reflects an early division in the *planitibia* subgroup that separates *picticornis* and *setosifrons* from the rest of the species [7].

The remaining 31 picture wing species in the database are divided into the *glabriapex* and *grimshawi* subgroups (Figs. 4, 5) based on the presence or absence of the 4b chromosomal inversion [40]. Most of these species have patterns that are variations on a basic plan of 7 spots: one at the distal tip of each longitudinal vein L2–5, a central spot on L4 at the posterior crossvein, a central spot on L2, and spot in the anterior/proximal region. This could be the ancestral pattern since it is found in the basal species, *glabriapex*. Most species also have an 8^th^ spot located centrally on L3, but this character has been gained or lost multiple times (based on the chromosomal lineage [40]). This L3 spot was gained at least once en route from the *glabriapex* to the *grimshawi* subgroup, then lost in *sproati* and *limitata*, and either gained or lost within the *orphnopeza*/*orthofascia* lineage. Fairly subtle variations in the intensity, extent, and position of these 7 or 8 spots can create very different visual effects: stripes in *hawaiiensis* and *orthofascia*, a “T” formation in *virgulata*, discrete spots in *discreta*, etc. The basic spot arrangement has been elaborated into an ornate checkerboard pattern in *grimshawi* and relatives, primarily by adding a proximal stripe along L3–4, and extending and refining the spot on L5. In *crucigera* the pattern is further shaped to form distinct crosses (as noted by Grimshaw in 1901 [34]) as well as two bulls-eyes in the posterior half. Comparison to the more basal *grimshawi* suggests that these isolated bulls-eye spots appeared *de novo* in clear areas of the pattern.

### Non-picture wing species groups

The *antopocerus* group species (Fig. 6, upper) are sexually dimorphic; males display long, specialized bristles on the foreleg, and extended aristae (visible on the *cognata* slide). The wings may be dimorphic in shape (*longiseta*) and pigmentation (*stigma*). The *stigma* wing pattern closely matches those of the Asian species *D. biarmipes* and *elegans*, which have been recently analyzed by Gompel et al. [25] and Yeh et al. [46].

The fungus feeder/*haleakalae* group (Fig. 6, lower) is the most basal lineage of the endemic Hawaiian *Drosophila* and diverged from the picture wing group an estimated 20 million years ago [10]. Some of these species are large, with relatively slender bodies and elongated wings; for example, *dolichotarsus* displays sexual dimorphism in which the male wing is quite extended and slightly curved (Fig. 6, lower left).

Fig. 7 shows *anomalipes* and *quasianomalipes*, which comprise the *anomalipes* group; they are closely related to the picture wings [8], [36]. The remaining samples represent the modified mouthpart [37] and modified tarsus [35] groups. These groups typically have plain wings, but exhibit remarkable male-specific specializations of the mouthparts or forelegs [47]. Light and SEM micrographs of some of these specializations will be presented elsewhere and added to the photo database.

### Photographic comparison of wing patterns

**Figure 8:**
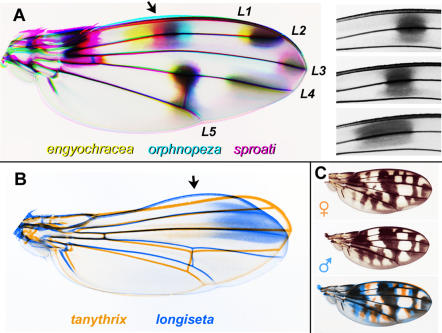
Analysis of pattern variation using color-coded overlays of wing photos. A. Pattern elements can vary independently. Left, *engyochracea* (yellow), *orphnopeza* (cyan), and *sproati* (magenta) are overlaid. Black indicates where all three coincide. Wings were uniformly skewed in Photoshop to maximize overlap of the margins and veins. Names of longitudinal veins are indicated; L1 is the costal or marginal vein. Arrow, the spot over L2 varies much more than the other spots. Right, positions of the variable spot on L2 are compared in the three species: upper, *orphnopeza*; middle, *sproati*; lower, *engyochracea*; the same region of the wing is shown in each case. B. Two specimens from the *antopocerus* group are overlaid: *tanythrix* (orange) and *longiseta* (blue). The wings were resized to overlay the anterior crossvein, L2 and L3, but the photos were not skewed. Arrow indicates where the anterior margin has a bump in *longiseta* but is concave in *tanythrix*. C. Sexual dimorphism in *cilifera*: a female (upper) and male (middle) are overlayed (lower; female in orange, male in blue). Wings were slightly rescaled to align the veins.

To better assess the variation among the wings, we made direct comparisons by color-coding sets of wing images and overlaying them (Fig. 8). In Fig. 8A, three *grimshawi* subgroup wings are overlaid: *engyochracea, orphnopeza*, and *sproati*. Among these species, the spot that occurs medially along L2 (arrow) can “slide” to different positions along the proximodistal axis, generating a rainbow-like pattern in the overlay; the other spots remain largely fixed. The proximal and distal borders of this spot can vary independently, as shown by the aligned close-ups of L2 (Fig. 8A, right). This result suggests that wing patterning genes somehow exert a very flexible, fine-scale control over the pigmentation process.

Species within one group can vary substantially in shape, as noted above for *truncipenna*. Fig. 8B shows another case, in which the anterior margin has shifted within the *antopocerus* group. *D. tanythrix* (not shown in Fig. 6) has a slightly concave anterior margin. *D. longiseta*, in contrast, has a bulge on the anterior margin (arrow). The overlay shows that the posterior compartment also differs, with L4 and L5 diverging strongly in *tanythrix*.

An overlay of male and female *cilifera* wings shows that sexual dimorphism is achieved by varying only a subset of pattern elements (Fig 8C). The large central spot and the distal-most spot are the same in both sexes (black), but the wave-like pattern in the posterior cell is shifted, and several spots are missing from the male (note the orange-only features).

### Pigmentation in mutants and natural variants

**Figure 9:**
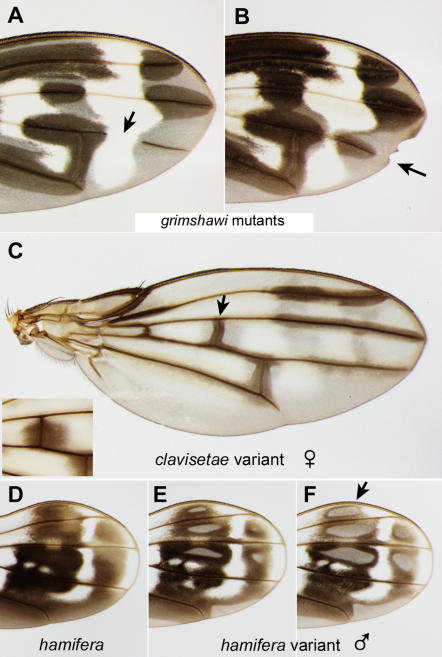
Variants that uncouple the prepattern from the vein-dependent pattern. A–B. Wings from *grimshawi* mutant lines. A. *weak veins* mutant with a gap in L4 (arrow); dark pigment is absent from the distal L4 vein fragment. B. Wing notching in a *Nihoa* mutant is associated with a shortened L4 and a reduced distal spot (arrow). In both A and B, the prepattern of dark hairs is not affected [21]. C. Rare, natural variant of *clavisetae* with incomplete pigmentation; this defect is seen in both wings. Compare the central crossvein in the variant (arrow) to that of a typical *clavisetae* female (inset). D–E. Wings from two *hamifera* males. D. Normal pattern. (This wing and the one in Fig. 2 are a pair from one male). E, F. Pair of wings from a different male. As in C, the intervein regions have not fully pigmented; see for example the spot indicated by the arrow.

Our database also includes several informative examples of wings that deviate from the standard pattern of the species (Fig. 9). Mutant lines of *grimshawi*, obtained by cobalt-60 irradiation, demonstrate that the integrity of the veins is essential for local melanization [21]. The recessive mutation *weak veins* causes a discontinuity in the vein (Fig. 9A, arrow), preventing pigment deposition distal to the break. The dominant wing notching mutation *Nihoa* leads to a shortened vein (Fig. 9B, arrow) that precisely coincides with the extent of pigment deposition there. These mutants consistently support the model that the pigmentation of the wing cuticle requires pigment precursors that are delivered through the veins to intervein regions. They also demonstrate that there is an inherent pattern of wing hair pigmentation that is not dependent on intact veins [21].

Natural variants are sometimes found in the field that also support this two-step model. Fig. 9C shows an unusual *clavisetae* wing in which the intervein spots did not become filled in; dark pigmentation is limited to narrow strips along the veins. The *clavisetae* prepattern is still apparent (grayish regions of the intervein territory). The wing pattern in *hamifera* can become altered in an even more complex manner. Compared to the typical pattern (Fig. 9D), the male in Fig. 9E–F has “holes” in five of the spots and incomplete pigmentation around L5. This male's two wings (panels E vs. F) differ in the extent to which the dark pigment has penetrated into the intervein regions, indicating the phenotype is sensitive to local conditions. One explanation is that this male did not produce enough pigment precursors in the body permit their complete transport/diffusion throughout the wing spots. This hypothesis is consistent with previous experimental findings of True et al. [21]: removing the wings from *D. rajasekari* upon eclosion prevents pigmentation from developing, but bathing these wings in the pigment precursor dopamine can restore the normal pigment pattern. Alternatively, in these *clavisetae* and *hamifera* examples, efficient transport could have been blocked by structural defects in the veins, extracellular matrix, etc.

### Possible drivers of wing diversity

Wing pigment patterns may be employed for courtship, camouflage, or mimicry [23], [26], [46], [48], [49], although their functions are difficult to prove experimentally. Without a firm grasp on their functional relevance in the wild, it is difficult to assess why the patterns have diversified so extensively. We speculate that, in many of the examples shown here, the patterns strike a balance between the need to hide from predators and the need to attract mates. At rest, when the wings are folded back over the thorax and abdomen, the wing patterns of many species blend with their dorsal cuticle markings, producing a camouflage pattern that could protect the fly from bird or insect predation [1]. The level of protection afforded by any given pattern could depend on a wide variety of environmental parameters that are unique to each species (and each sex within a species). For example, females spend considerable time seeking favored substrates for oviposition; one species may need to blend in with bark, another with leaves, etc. [1], [8].

During courtship, however, males of many picture wing species prominently display their wing markings to the female. The female might use these markings for species recognition and even to assess the fitness of the male: we have noted that smaller flies tend to have noticeably reduced pigment spots. Sexual selection is known to be a key driver of morphological change in the Hawaiian flies [3], [27], [47]. Several species groups are characterized by extreme male-specific ornamentations used to stimulate the female during courtship, including major modifications of the forelegs, bristles, antennal structures, and mouthparts. Altering wing pigmentation would seem to have a lower fitness cost than these other options, and may be favored for that reason. On the other hand, female flies may not be as attuned to visual cues as to tactile ones. Finally, the Hawaiian species have generally been subject to small population sizes and frequent exposure to founder events and bottlenecks. Over time, a given lineage accumulates a unique set of random mutations in the pigmentation genes. Essentially, each species is dealt a different genetic hand that it can use to accommodate these diverse selection pressures, and this may also contribute to diversification.

### Hawaiian *Drosophila* as a model for the evolution of complexity?

We show several examples in which, along a known lineage, species exhibit increasing pigment pattern complexity or gain/loss of discrete pattern elements. It will be extremely informative to sequence candidate loci such as *yellow* and *ebony* in these species. *D. primaeva* provides a convenient reference species since it has no spots on the longitudinal veins, and is the most basal picture wing species; presumably both the plain wing pattern and the sequence of the pigmentation genes are fair representations of the ancestral state of this group (Fig. 1). The complex including *villosipedis, grimshawi*, and *crucigera* provides a clear example of increasing complexity (Fig. 1). These three species are similar or identical at the polytene chromosome level, and *grimshawi* and *crucigera* genes differ by just one base change or small indel every 55 bp (averaging over the 6 *crucigera* nuclear genes present in Genbank). Thus, comparisons among these species could provide insights into the evolution of complexity.

Another candidate for comparative study is the *adiastola* subgroup. Evidence suggests the basal *primaeva* wing gave rise to the simple, wave-like pattern of *ornata*, and the more derived species have extensively modified this pattern along different branches of the *adiastola* subgroup (Figs. 1, 2). We would expect to find shared mutations that are responsible for shared pattern elements, and additional mutations that differ in each branch of the lineage and account for novelties in the pattern [26]. Functional mutations identified in one subgroup can then be compared to other subgroups that have qualitatively different types of patterns; for example the *grimshawi* subgroup is characterized by distal spots, while the basal species of the *adiastola* subgroup lack distal spots. This approach would capitalize on a rare advantage of the Hawaiian *Drosophila*, that pattern evolution has been “replayed” multiple times in a shared genetic background.

### Are the Hawaiian *Drosophila* tractable for developmental biology?

The utility of the Hawaiian flies for experimental studies varies substantially among species. We can consider several levels of experimental tractability relevant to the studies suggested above: (1) availability of genomic DNA for comparative sequence analysis; (2) ability to grow larvae for studies of gene expression and developmental biology; (3) ability to make transgenic flies; (4) ease of performing transmission and quantitative genetics (keeping multiple lines, generating markers, obtaining fertile hybrids, etc.)

Each of these milestones has been reached in the picture wing flies, albeit with more effort than required for *D. melanogaster*. DNA is available from most of the species pictured here, and cloning genes of interest will be greatly facilitated by the high sequence identity levels among the Hawaiian species. Carson's chromosome phylogeny was derived by analysis of larval chromosomes, indicating that larvae can be cultured from nearly every picture wing species [43]. We have successfully performed immunostaining of picture wing larvae and pupae using several antibodies to *D. melanogaster* proteins (not shown). Transgenic Hawaiian Drosophila have been produced by injecting P element DNA into *D. hawaiiensis* embryos [50]. There were no visible markers available at that time, so transformants were identified by Southern blot analysis of the offspring of individual injected animals. Current availability of additional transposon vectors and transformation markers should simplify the transformation process [51]. It should be possible to transform flies with both plain (*mimica*) and ornate (*grimshawi, crucigera*) wing patterns, although *grimshawi* lay eggs at a greater rate than *mimica*. For optimal egg collection, specialized substrates are required (e.g., moistened corn flakes.) Stocks of *mimica, grimshawi*, and several other endemic Hawaiian species are available at the Tucson Drosophila Species Stock Center. Genetic markers are not currently available, although we have demonstrated that visible mutations can be isolated and maintained in *grimshawi* [21]. The greatest limitations to genetic analysis are the space and labor required for stock keeping (see Materials and Methods), and the 2–3 month generation time. Finally, it should be possible to identify X chromosomal vs. autosomal contributions to patterning, and estimate the number of major loci involved, by hybridizing species with distinct wing patterns in the lab (as done for *silvestris* vs. *heteroneura* coloration and head shape [52]). *D. grimshawi*, for example, can hybridize with *balioptera, bostrycha, crucigera, disjuncta, pilimana*, and others [53].

## Materials and Methods

### Field collections

Flies were collected from banana or mushroom baits, or by netting, at previously described locations on Kauai, Oahu, Molokai, Maui, and Hawaii (Big Island) [1], [36], [43], [54]. Species identifications were made by K.Y.K.

### Stock maintenance

For *disjuncta, grimshawi, hemipeza, heteroneura, planitibia*, and *silvestris*, specimens were taken from laboratory stocks maintained at the Univ. of Hawaii at Manoa instead of from the field. Picture wing stocks are cultured at 18°C. Oviposition occurs in vials of Wheeler-Clayton medium [55] supplemented with an aqueous extract of *Clermontia* (a natural host plant which helps to stimulate oviposition). Once larval activity is observed, cornmeal-molasses-agar medium is added to the vials. Vials with third instar larvae are placed in a gallon jar half filled with damp, coarse sand. Larvae tunnel into the sand to pupate, and adults crawl back out upon eclosion. Newly eclosed adults require 2–3 weeks to reach sexual maturity; females especially require 3–4 weeks before mating and egg laying begins. Temperature and humidity regulation, culture media specific to larval and adult nutritional requirements, sterile sand as the pupation medium, etc., make laboratory husbandry of the Hawaiian *Drosophila* species significantly more complex than *D. melanogaster*. However, a number of laboratories in the U.S. as well as internationally have been successful in maintaining laboratory stocks of Hawaiian *Drosophila* and have been able to conduct genetic and behavioral studies on these species.

### Sample preparation and documentation

In most cases wings were removed from live flies; for *algaia, cyrtoloma, clydonia, differens, diminuens, hamifera* female, *hirtipalpus, truncipenna*, and *virgulata*, the flies were recently dead when the wings were removed. Older, pinned specimens were found to be rather unsuitable for the project since their pigmentation had faded. The wings were permanently mounted in Euparal (BioQuip Products, Gardena, CA) between a slide and coverslip, taking care to avoid damage and folding. Slides were incubated overnight at 37°C to allow bubbles to dissipate, and stored in the dark. The wings were all photographed in one session under uniform conditions, using a digital camera mounted on a dissecting scope and illuminated with an overhead ring light. Raw images were adjusted in Photoshop using the “Warming Filter 81” command to neutralize the background toward gray, and contrast was restored using “Curves”. All adjustments were performed to make the backgrounds uniform across images, so that the wings are as directly comparable as possible. In Figs. 2–7, debris was edited out of some photos using Photoshop, but the wings were not altered; the unedited photos are found in the database. In Fig. 2 and Fig. 5, the backgrounds (away from the wings) were blurred to facilitate file compression. All images in the database were taken at the same magnification. In Figs. 2–7, all wings in a given figure are on the same scale, so one scale bar is shown per figure. The sex is listed if known. Only in Fig. 8, some wings were distorted using the “scale” or “skew” commands where noted. See Stark et al. [13] for explanation of wing vein nomenclature; the *Drosophila* system is used here for simplicity and longitudinal veins L1–5 are defined in Fig. 8A.
